# From protection to pollution: Evaluating environmental and human health risks of acaricide use in dairy farming in Kenya

**DOI:** 10.1371/journal.pone.0333694

**Published:** 2025-10-17

**Authors:** Kevin W. Maina, Martin C. Parlasca, Elizaphan E.J.O. Rao

**Affiliations:** 1 Center for Development Research (ZEF), University of Bonn, Bonn, Germany; 2 International Livestock Research Institute (ILRI), Nairobi, Kenya; Beni-Suef University, EGYPT

## Abstract

Sustainable intensification of livestock production relies critically on effective disease management, yet the environmental implications of current practices remain poorly understood. The study was designed to evaluate the efficacy of acaricide use in tick control in Kenya’s dairy sector affects environmental and human health risks. Using original survey data from dairy farmers and a two-stage least square (2SLS) approach, the results found that farmers’ adaptation to perceived ineffective tick treatment leads to potentially harmful practices. Twenty percent of farmers improperly rotate acaricides, while 66% under-apply recommended doses. Despite 65% using protective gear, 29% report adverse health effects. Our estimates show that improper acaricide group rotation increases the environmental and human health risks by 35%. The study highlights important trade-offs between animal health management and environmental and human health objectives, suggesting a need to reform current disease prevention approaches to balance productivity gains with environmental sustainability in developing countries.

## Introduction

Chemical pesticides remain integral to modern agricultural production, offering critical benefits in crop protection and livestock disease management. In developing countries, where agricultural productivity gains are central to economic transformation, the continued use of pesticides has raised mounting concerns about environmental degradation, biodiversity loss, and human health risks from chemical exposure [1–3]. These environmental and human health trade-offs are salient in livestock systems, where farmers regularly apply synthetic acaricides to control ticks.

In Sub-Saharan Africa (SSA), where tick-borne diseases like East Coast Fever (ECF) have been shown to cause considerable losses in productivity due to morbidity and mortality [4,5], weekly chemical treatments through spraying or dipping animals have become standard practice [6]. Yet, the effectiveness of these chemical controls is increasingly compromised by growing tick resistance linked to the frequency of acaricide change, herein referred to as acaricide active ingredient group rotation. Failure to adhere to recommended acaricide rotation practices has been shown to increase tick resistance over time [7,8], often prompting farmers to adopt potentially harmful practices like increased application frequency or admixing of different chemical products to increase perceived effectiveness in tick control [9,10].

In the current study, the environmental and human health implications of acaricide use were examined in Kenya’s dairy sector, where intensive tick control is critical for maintaining productive cattle herds. Using original survey data from 412 dairy farmers, the study analyzes current acaricide application practices and their association with environmental and health risks.

In Kenya, dairy production is primarily classified into three systems namely extensive grazing systems, semi-intensive, and intensive systems [11]. Intensive systems are characterized by adopting high-yielding exotic breeds of cattle that are zero-grazed mainly in regions with small-landholding and high population density around Central Kenya and peri-urban areas of the capital Nairobi. Semi-intensive systems are characterized by low adoption of high-yielding breeds with farmers practicing semi-confined systems that combine grazing and stall feeding on largely unimproved fodder [12]. This forms part of the Western region in Kenya including the North Rift region. These production systems face challenges from tick infestation with the risk of cows of improved breeds being susceptible to ECF [13].

Despite the existence of alternative vector control approaches, chemical control using acaricides remains the primary mode of tick control for most farmers [9,14]. However, studies show current chemical tick control approaches have experienced increased incidences of acaricide resistance [8,15]. This is further exacerbated by farmers’ limited knowledge of proper acaricide application practices [7,10]. To successfully control ticks using acaricides, farmers are required to adhere to instructions on application rates per animal, a correct mixing ratio of acaricides and water, and recommended acaricide chemical group rotation – which involves changes in acaricides of different chemical groups based on active ingredients (AI) to avert tick resistance [16].

Acaricide rotation is meant to reduce the development of tick resistance whereby farmers switch between different active ingredient groups over a specified interval (number of months). Despite no consensus on the optimal number of months to consider for acaricide group rotation [15], studies show a 6–12 months interval – approximately 2 acaricide groups annually – to be an appropriate rotation period in African livestock systems [10,17]. However, what remains unknown is whether farmers adhere to these recommendations and how their practices affect the effectiveness of tick control.

Improper acaricide group rotation over time often leads to acaricide failure, prompting farmers to switch between acaricides at shorter intervals, increasing the number of acaricide products used [18]. Farmers may also engage in other unsafe acaricide application practices, such as applying increasingly higher chemical dosages beyond recommended rates or hazardous mixing of acaricides with other groups of pesticides [15,19]. Thus, improper acaricide group rotation is likely to be correlated with an increased number of acaricides used by farmers. Consequently, this has potentially negative implications on the environment and human health risks due to contamination and exposure of soil, water, and animal products by chemical residues over time [20–22].

However, these effects may be context-specific, and empirical literature in the context of livestock systems remains scarce. Therefore, our analysis is explorative and aims to investigate the association between improper acaricide group rotation practices and potential environmental and human health risks measured by EIQ.

## Materials and methods

### Ethics statement

The study received ethical approval from the Centre for Development Research (ZEF), University of Bonn research ethics board, clearance reference 8b_22 Kevin Maina. The study obtained written informed consent from all the respondents.

### Data

A farm household survey was conducted in Elgeyo Marakwet, Uasin Gishu, and Nandi Counties to understand acaricide use and its environmental implications. A multistage sampling technique was followed to select farmers. In the first stage, based on active membership, 5 dairy cooperatives (Ainabkoi, Chepkorio, Lessos, Lelelchego, and Tarakwo) were purposively selected. In the second stage, 64 milk collection clusters with a minimum of 20 (This is the least number of sample size per cluster that can allow random replacements.) farmers were randomly selected. The third stage involved a random selection of 49 clusters and 578 dairy farmers using proportionate random sampling.

Face-to-face interviews were held in October-November 2023 with either the household head or the spouse. Our findings showed 71% of the sampled farmers use hand spraying as their main method for tick control, compared to 29% who use dipping (both private and public) as the main method for tick control. For our analysis, we focus on farmers who use hand-spraying as their main method for tick control further reducing our final sample to 412 dairy farmers. This was informed by two main reasons. First, from an analytical perspective, it is difficult to quantify the parameters we use in estimating the EIQ from acaricides used in cattle dips. Second, farmers may lack completeness of data relating to the type of acaricide used in the cattle dip and the amounts applied.

Most sampled households are male-headed with an average of 21 years of dairy farming experience (Table 1). The household heads mainly practice farming as their main occupation (75%). The average herd size for dairy cattle is about 5 tropical livestock units (TLU), translating to farmers keeping an average of 5 heads of cattle. However, only 35% of the farms practice grazing systems implying a higher proportion of farms under zero-grazed intensive production systems.

**Table 1:** Descriptive statistics of sampled farmers.

Variables	Mean	Std dev
Male household head (male = 1)	0.80	
Dairy farming experience (years)	21.08	14.00
Household head main occupation (farming = 1)	0.75	
Grazing system (yes = 1)	0.35	
Livestock ownership (TLU)	4.95	3.87
Log annual household expenditure (KES)	12.45	1.21
Number of extension visits (annually)	4.37	14.01
Distance to nearest vet shop (kilometers)	2.74	1.85

Notes: N = 412. TLU = tropical livestock units with conversion factors based on [23] for Sub-Saharan Africa.

### Measuring the effects of acaricide use on the environment and human health risks

Assessing the changes in the frequency of application of acaricide or application rates per animal due to changes in the incidence of improper acaricide group rotation can be useful indicators of reduced risks to the environment and human health. However, this approach does not consider differences in specific acaricides used by farmers that may have varying environmental and human health risks. Chemical pesticide products differ in terms of toxicity levels and persistence [24]. To overcome this challenge, the study used the environmental impact quotient (EIQ) developed by [25] to measure the environmental and human health risks associated with acaricide use. This approach relies on toxicological information on different chemical products to give a single numerical indicator of the risks to farmer workers, consumers, and the environment [26]. Despite criticism of the use of arbitrary weights, EIQ proves useful as a proxy to comprehensively measure environmental and human health risks in the absence of alternatives. It has been used in different contexts to estimate the effects of pesticides in cropping systems [24,27–29].

We first extract data on active ingredients (AI) for each acaricide product used by farmers and further collate the EIQ values for each of the AI from Cornell University College of Agriculture and Life Sciences (https://cals.cornell.edu/new-york-state-integrated-pest-management/risk-assessment/eiq/eiq-pesticide-values) database. We present EIQ values for different acaricides being used by farmers in S1 and S2 tables in S1 File in the online supplementary material. Following [24] and [26] the study makes a slight alteration to EIQ field use computation that allows for comparisons. The EIQ field use is given by:


EIQ field use=EIQ × % active ingredient×frequency of application×dose or application rate (1)

Application rate/dose in cropping systems is given by liters/ha or kg/ha, in our case we consider liters per animal as a measure in acaricide application [10].

### Estimation strategy

The relationship between improper acaricide group rotation and environmental and human health risks was estimated using the following specification:


$$Y_{i}=\beta_{\text{0}}+\beta_{\text{1}}R_{i}+\beta_{\text{2}}X_{i}+\varepsilon_{i}$$ (2)

where $Y_{i}$ represents EIQ for household i, capturing the environmental and human health effects of acaricide use, $R_{i}$ is a dummy variable measuring improper acaricide group rotation. $X_{i}$ is a vector household socio-demographic characteristics, and $\varepsilon_{i}$ is the error term. The parameter of interest, $\beta_{\text{1}}$, captures the marginal effect of acaricide usage on environmental and health risks.

While equation (2) provides a baseline relationship, OLS estimation likely yields biased estimates of $\beta_{\text{1}}$ due to endogenous selection in acaricide usage. Farmers’ decisions regarding the frequency and type of acaricides are potentially correlated with unobservable characteristics such as managerial ability, risk preferences, and access to institutional resources. For instance, more skilled farmers might optimize their acaricide rotation strategies while simultaneously implementing other practices that affect environmental outcomes. Similarly, risk-averse farmers may both over-apply acaricides and take other precautionary measures that influence the EIQ. These selection issues could bias our OLS estimates in either direction.

To address these endogeneity concerns, we depict this as a causal chain and employ a two-stage least square (2SLS) approach. We instrument the total number of acaricide products used using the incidence of improper acaricide group rotation. The validity of our identification strategy rests on two key conditions. First, regarding relevance assumption, improper acaricide group rotation should strongly predict overall acaricide usage – the total number of acaricide products farmers use. The first-stage results in Table 6 confirm this relationship, with an *F*-statistic of 19.09, well above the [30] threshold of 10 for weak instrument concerns.

**Table 6:** First-stage regression result on improper acaricide rotation and the number of acaricides used annually.

	Number of acaricides used annually	
	(1)	(2)
Incidence of improper acaricide rotation (yes = 1)	0.21**	1.34***
	(0.07)	(0.11)
Constant	7.12***	1.74***
	(0.18)	(0.42)
Controls	No	Yes
R-squared	0.07	0.32
Observations	412	412
F-Statistic	8.27	19.09

Notes: Coefficients are shown with robust standard errors in parentheses. Controls include gender, dairy farming experience, main occupation of the household head, livestock management system, herd size (TLU), household expenditure, number of extension visits annually, use of protective gear when spraying, and distance to a veterinary shop. Statistical significance at *p < 0.1, **p < 0.05, ***p < 0.01.

Second, our exclusion assumption requires that improper acaricide group rotation affects environmental impact only through its influence on the total number of acaricides used. While this assumption is inherently untestable [31], the study argues for its plausibility based on the institutional context of acaricide purchases. Most farmers lack detailed knowledge of active ingredients when making purchasing decisions, effectively randomizing the proper/improper nature of their rotation sequences. This information gap creates quasi-random variation in rotation quality that is plausibly exogenous to unobserved determinants of environmental impact.

In addition, a sensitivity analysis for the 2SLS estimation was performed using the kinky least squares (KLS) regression. Kinky least squares (KLS) is an instrument-free model that overcomes challenges associated with IV approaches [32]. The graphical outputs from the approach allow us to compare the confidence intervals for both KLS and IV, providing insights into the strength of our instrument. Weak instruments are associated with wider confidence intervals from the IV approach compared to the Kinky approach [33].

We proceed with the 2SLS strategy as follows:


$${First\;stage:\;R}_{i}=\beta_{\text{0}}+\beta_{\text{1}}S_{i}+\beta_{\text{2}}X_{i}+\varepsilon_{i}$$ (3)

$${Second\;stage:\;Y}_{i}=\beta_{\text{0}}+\sigma_{i}{\hat{R}}_{i}+\beta_{\text{2}}X_{i}+\varepsilon_{i}$$ (4)

where $S_{i}$ in the first stage measures the effect of improper acaricide group rotation in household i’s acaricide rotation sequence on the number of acaricide products used, and ${\hat{R}}_{i}$ in the second stage represents the predicted values from the first stage. The controls, $X_{i}$ are as defined in Equation 2.

## Results

### Acaricides used in dairy farms in Kenya

**Fig 1:**
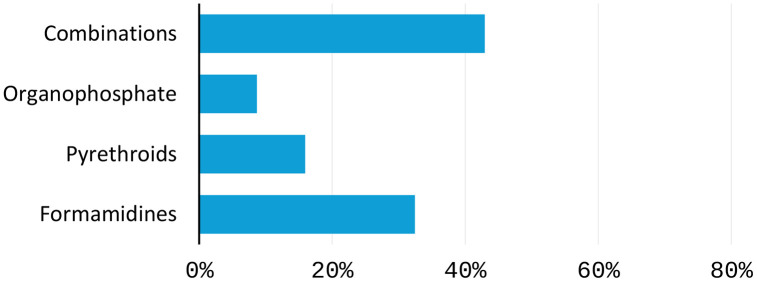
Acaricide groups used by dairy farmers. Multiple answers were possible. N = 412.

Fig 1 and Table 2 summarize the different acaricide groups and products used in the control of ticks by sampled farmers. On average farmers apply acaricides three times a month, translating to a seven-day-interval between sprays on average. Farmers use a particular acaricide product for six months before switching to a different acaricide group. This means that, on average, farmers use two different acaricides annually.

**Table 2:** Common acaricides used by farmers for tick control.

Acaricide group	Active ingredient (AI)	% for each active ingredient	Trade name		Number of households using	Number of applications per month in the last one year	Number of months used in the last one year	WHO hazard classification
Formamidines	Amitraz 12.5%	32.07		Actraz®	3	2.33 (1.53)	3.67 (2.08)	II
				Almatix®	13	2.69 (1.18)	5 (3.37)	II
				ByeBye®	2	1.5 (0.71)	4 (2.83)	II
				Norotraz®	40	3.1 (1.15)	5.42 (3.43)	II
				Taktic®	13	2.69 (1.25)	4.77 (2.71)	II
				Tikfix®	14	2.71 (1.33)	5.07 (3.81)	II
				Triatix®	216	2.79 (1.21)	6.38 (3.49)	II
				Twigatraz®	3	3 (1.73)	8 (3.46)	II
Pyrethroids	Alpha-cypermethrin	4.22		Cypertix	38	3.18 (1.18)	4.76 (3.13)	II
				Daltix	1	2	4	II
				Eliminator	1	4	6	II
	Lambda-cyhalothrin	0.11		^a^Duduthrin®	1	2	12	II
	Cyhalothrin 5%	4.11		Grenade	39	2.90 (1.17)	4.74 (2.37)	II
	Cypermethrin	1.48		^b^Cypertex	1	4	2	II
				Ectomin	13	2.54 (1.33)	5.15 (3.56)	II
	Deltamethrin	6.43		Delete	61	2.67 (1.47)	5.77 (3.70)	II
Organophosphate	Chlorfenvinphos	8.76		Steladone	83	3.07 (1.18)	5.14 (3.17)	Ib
**Acaricide group**	**Active ingredient (AI)**	**% for each active ingredient**	**Trade name**		**Number of households using**	**Number of applications per month in the last one year**	**Number of months used in the last one year**	**WHO hazard classification**
Combination	Chlorpyrifos 50% + Cypermethrin 5%	40.51		Cyperdip®	11	3	6	II
				Dabotik®	8	2.29 (1.25)	7.86 (4.01)	II
				DuoDip®	334	2.84 (1.27)	7.19 (3.52)	II
				Pyrotix®	1	4	2	II
				Sidai Ultradip®	18	3.06 (1.30)	4.82 (2.96)	II
				TikDip®	9	2.78 (1.48)	6.67 (4.09)	II
				Ultradip®	3	1.67 (0.58)	6.33 (4.93)	II
	Cypermethrin + Chlorpyrifos + Piperonyl butoxide + Citronell	2.32		Vectoclor®	22	3.04 (1.76)	6.86 (3.73)	II

Notes: Standard deviations in parentheses. a = insecticide being used in tick control; b = crop pesticides being used in tick control; World Health Organization (WHO) pesticide hazard classification of pesticide active ingredient: Ib = highly hazardous, II = moderately hazardous, III = slightly hazardous, U = unlikely.

Our findings show a significant inverse correlation (−0.35) between the duration of acaricide use and the frequency of its monthly application. This suggests that when farmers use an acaricide product for a longer duration, they tend to spray less frequently each month. A plausible explanation is that more effective acaricides provide better tick control, reducing the need for frequent spraying (i.e., every seven days). In contrast, when acaricides are less effective, farmers may need to apply them more often or switch to different products to achieve better results. Most farmers use acaricides with combined active ingredient, mainly a combination of cypermethrin and chlorpyrifos (Table 2). They also use acaricide products in the formamidines group where the main AI is *amitraz*. We also observe cases of farmers using other chemicals classified as pyrethroids to control ticks, for example, Duduthrin® and Cypertex products are mainly used to control crop pests such as aphids and armyworms in crops. Further qualitative probing from farmers on the use of crop pesticides indicates that the effectiveness is perceived to be higher in tick control than the available acaricides classes in the region.

Based on the [34] hazard classification of pesticides, most of the acaricides in use are classified as moderately hazardous (II) except for Steladone which is classified as highly hazardous (1b) (Table 2).

### Acaricides application practices and potential human health risks

Table 3 summarizes acaricide application practices among sampled farmers. We find that 20% of farmers improperly rotate their acaricides. This involves switching acaricides within the same acaricide group and is likely to cause the build-up of tick resistance over time [16].

**Table 3:** Acaricide application practice among farmers.

	Percentage
***Acaracide AI group rotation***	
Incidence of improper acaricide rotation (1 = yes)	20.39
***Acaracide usage based on application rate per animal (recommended 5 liters of diluted solution per animal)***	
Under application	65.67
Recommended application	14.48
Over application	19.85

Notes: N = 412.

Additionally, 66% of farmers tend to under-apply acaricides on animals and are likely to increase the risk of tick infestation and acaricide failure [14,15]. The recommended application rate of a diluted acaricide solution is 5 liters per animal per spray [10]. About 20% of farmers apply acaricides above the optimum recommended levels and only 14% of farmers applying the recommended level.

Table 4 summarizes acaricide measurement practices among farmers. On average, most farmers use measuring cylinders to achieve the correct acaricide dilution ratio. Most acaricides are sold with calibrated measuring cylinders and the dilution ratio is provided on the packaging. We do not observe differences in proportions between farmers who follow proper acaricide class rotation and those who follow otherwise. This in part shows that most farmers have some level of knowledge on dilution ratio and can follow manufacturer’s instructions.

**Table 4:** Acaricide measurement practices among farmers.

Measurement practices	Percentage
Measuring cylinder	86.17
Bottle top	12.14
Use of eyes to estimate	1.70

Notes: N = 412.

However, we observe some farmers using bottle tops to estimate the acaricide dilution ratio. Most times farmers use the bottle tops of the acaricide product, thereby relying on an estimate as opposed to measuring the exact dilution ratio. While this may be considered a bad practice, qualitative insights from the farmers show that with sufficient experience, one can measure accurately recommended amounts similar to using calibrated cylinders.

**Fig 2:**
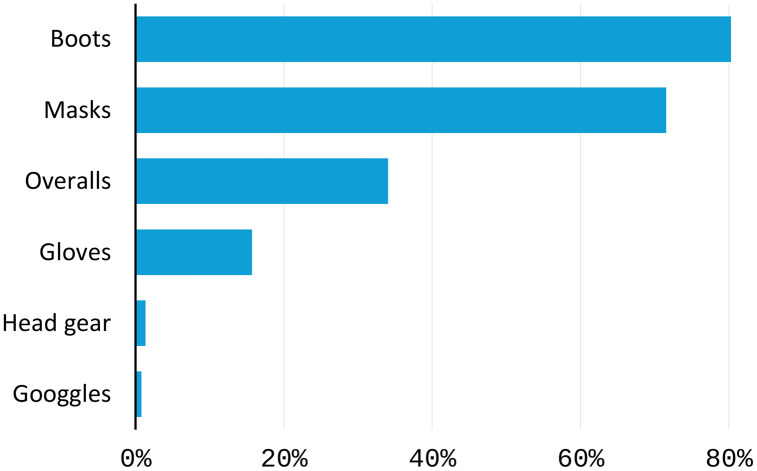
Use of protective gear in the application of acaricides. Multiple answers were possible. N = 412.

The study finds that 65% of sampled farmers use some form of protective gear when applying acaricides. Fig 2 presents results on different forms of protective gear when handling and applying acaricides. This implies that farmers are aware of human health risks associated with acaricide use and follow recommendations to reduce exposure. The personal protective materials include boots, overalls, nose masks, gloves, and goggles to prevent adverse effects from exposure to acaricides.

Despite farmers using some form of protective material, the study found reported cases of adverse effects from acaricides (29%), as shown in Table 5.

**Table 5:** Reported cases of adverse effects of acaricide after application and symptoms.

	Percentage
***Adverse effects from acaricide use***	
Incidence of adverse effects from acaricide use (yes = 1)	29
***Household members affected***	
HH head	53
Spouse	19
Child	22
Farm worker	6
***Type of symptoms (symptoms consistent with intoxication)***	
Headache	30
Sneezing	24
Irritation in the eyes	26
Dizziness	23
Nausea	23
Shortness of breath	6
Skin irritation	23
Fatigue	2.89

Notes: N = 412**.** Multiple answers were possible.

Adverse effects from acaricide were mainly experienced by the household head with symptoms including headaches, irritation in the eyes, sneezing, dizziness, nausea, and irritation of the skin. These symptoms are consistent with the level of exposure, especially among the farmers who do not use any form of protective gear.

### Regression results

Table 6 presents the first-stage regression results, examining the relationship between improper acaricide group rotation and the total number of acaricide products used. The estimates reveal a strong and statistically significant relationship supporting the relevance condition of our identification strategy.

Column (1) presents the baseline specification without controls, while Column (2) includes our full set of household and farm-level controls. The coefficient on improper rotation increases substantially from 0.21 to 1.34 (p < 0.01) when including controls, suggesting that observable characteristics play an important role in mediating the relationship between rotation practices and overall acaricide use. This finding indicates that farmers who improperly rotate active ingredients use, on average, 1.34 more acaricide products annually compared to those who follow proper acaricide rotation protocols.

The magnitude of this effect is economically significant, representing approximately 67% of the mean number of annual acaricides that farmers use in our sample. The positive coefficient aligns with our theoretical expectations: improper rotation practices likely lead to reduced acaricide treatment effectiveness, compelling farmers to increase the frequency of application or experiment with additional products to maintain tick control.

These first-stage results yield important implications for understanding farmer behavior and agricultural extension services. The strong positive relationship suggests that inadequate knowledge of active ingredients leads to inefficient pest management practices, resulting in increased chemical use. The substantial change in the coefficient magnitude when including controls indicates that socioeconomic and farm characteristics significantly influence acaricide management decisions.

Next, our main results examining the relationship between the total number of acaricide products and environmental and human health impact, as measured by the environmental impact quotient (EIQ) field use value, are reported in Table 7. Both OLS and instrumental variable estimates were reported, with the latter addressing potential endogeneity in farmers’ acaricide rotation decisions.

**Table 7:** 2SLS estimates of the association between the number of acaricides used annually and annual EIQ field use.

	Log annual EIQ field use	
	(1)	(2)
Number of acaricides used annually	0.29***	0.26***
	(0.05)	(0.08)
Constant	8.91***	8.95***
	(0.66)	(0.67)
Controls	Yes	Yes
R-squared	0.16	0.16
Observations	412	412

Notes: Coefficients are shown with robust standard errors in parentheses. Controls include gender, dairy farming experience, main occupation of the household head, livestock management system, herd size (TLU), household expenditure, number of extension visits annually, use of protective gear when spraying, and distance to a veterinary shop. Statistical significance at *p < 0.1, **p < 0.05, ***p < 0.01.

The OLS estimates in column (1) indicate a significant positive association between acaricide usage and environmental and human health risks, with each additional acaricide product used associated with a 29% increase in the EIQ field use value (p < 0.01). Our preferred 2SLS specification in column (2), which instruments for improper rotation practices, yields a similar but slightly smaller coefficient of 0.26 (p < 0.01). This suggests that each additional acaricide product due to improper acaricide rotation increases the environmental and health risk indicator by 26%, holding other factors constant. Therefore, the incidence of improper acaricide group rotation is likely to increase the EIQ value by 35% (Computed by multiplying first-stage and second-stage coefficients (1.34 and 0.26, respectively)). The similarity between OLS and 2SLS estimates suggests that selection bias may not be severely distorting the relationship between acaricide use and environmental human health impact in our context. Moreover, the similarity also suggests that observable characteristics might be useful in identifying farmers at higher risk of engaging in environmentally damaging pest management practices.

The magnitude of these effects is economically significant. Given the mean annual EIQ field use value in our sample of 256, this implies that each additional acaricide increases the potential environmental and human health risks by approximately 66.62 units annually. The persistence of this large effect in our 2SLS specification provides robust evidence that intensive acaricide rotation practices substantially amplify environmental and human health risks.

### Robustness checks

**Fig 3:**
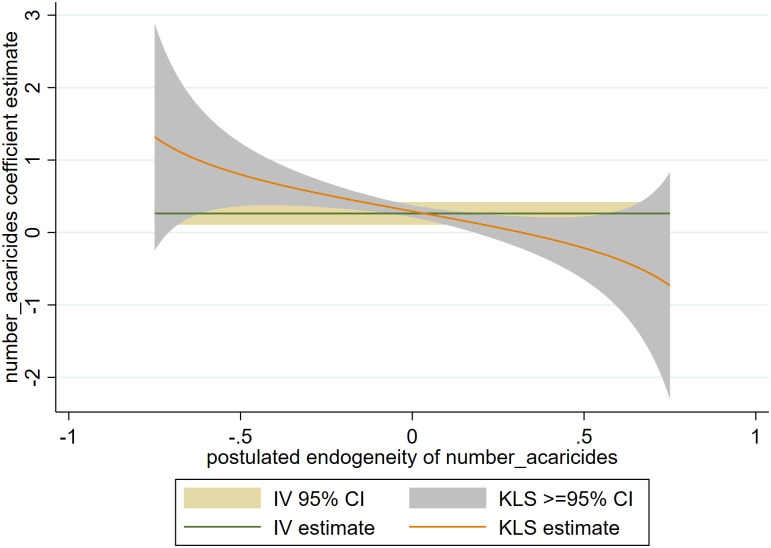
2SLS and KLS coefficient estimates and confidence intervals.

Robustness/ sensitivity checks on our 2SLS estimation was performed using the Kinky Least Square regression. Here, a comparison of the confidence intervals from the 2SLS approach and KLS is drawn to assess the weakness of our selected estimation. As shown in Fig 3, we find that the confidence intervals for our 2SLS approach are not wide validating our estimations. Additionally, we observe an overlap in the 2SLS and KLS estimation supporting the validity of our approach.

These observations imply that our estimates are unlikely biased. However, our study may not have adequately controlled some unobserved heterogeneity. Thus, we interpret our findings with caution.

## Discussion

Farmers’ response to pest infestation is driven by risk perception, access to advisory services, and other economic drivers. The results from this study show that while farmers generally use recommended acaricides, there is still widespread suboptimal acaricide application practices and risky adaptations. Descriptively, the study finds farmers engaging in admixing acaricides with other pesticides/insecticides for crops to increase efficacy. Even with 65% of users wearing protective gear, nearly 30% reported experiencing varying health effects, suggesting potential unsafe application practices. Moreover, farmers also engage in improper acaricide rotation practices, potentially contributing to acaricide resistance that can amplify the cycle of farmers engaging in malpractices in acaricide use. These patterns reflect knowledge and resource constraints among farmers. These results are not surprising as similar improper pesticide use practices have been documented in both crop and livestock production in Africa and Asia [1,9,10].

Further, the results showed that improper active ingredient rotation significantly increases environmental and human health risks – an incidence of improper active ingredient rotation in a farmer’s annual rotation increases the environmental and human health risks, as measured by the environmental impact quotient (EIQ), by 35%. Farmers who over-rotate, on the other hand, are likely using acaricides perceived to have more efficacy and be more effective in controlling ticks. While these farmers can effectively control ticks, in the long run, they are likely to have an incidence of tick resistance as a result of over-use of one acaricide [8,15].

These findings illuminate several important mechanisms in the complex relationship between livestock health management and environmental and human health outcomes. First, they suggest a concerning feedback loop in tick control practices: as documented by [9] in Uganda, improper rotation of acaricides often leads to tick resistance, compelling farmers to increase application frequency and switch to acaricides perceived to be more effective. A review by [18] also highlights this concerning feedback loop among farmers calling for novel acaricides and regular monitoring to avert resistance. Similar to previous studies in crops [35], lack of knowledge among farmers may perpetuate this cycle, resulting in overuse and underuse of pesticides.

Our results indicate that this adaptive behaviour significantly amplifies potential environmental and human health risks. The magnitude of our estimates suggests that the environmental and human health costs of these adaptation strategies may be substantial and previously underappreciated in the literature. [16] in their studies in Zambia and Burkina Faso highlight that it is also possible for alternative tick management regimes to avert these environmental and human health costs.

Second, our findings highlight the environmental and human health implications of farmers’ behavioural responses to perceived treatment inefficacy. Descriptive evidence, as discussed earlier, reveals that farmers often resort to potentially harmful practices such as admixing acaricides with other pesticides to enhance efficacy. These empirical results support previous findings where farmers engage in potentially harmful practices to improve the efficacy of chemicals [10]. Reconciling this descriptive evidence with 2SLS results suggests that these compensatory behaviours may be driving the environmental and human health impact beyond the direct effects of increased frequency in acaricide rotation. This points to an important interaction between farmers’ technical knowledge, pest management decisions, and environmental outcomes [2]. Altogether, the substantial environmental costs we document suggest that interventions targeting proper acaricide rotation practices could yield significant environmental and human health co-benefits alongside their primary goal of improving animal health.

Finally, our analysis contributes to three strands of literature. First, we add to research on agricultural intensification, and environmental and human health in developing countries [36–38]. While extensive work has documented the environmental impacts of pesticide use in crop production, far less attention has been paid to livestock systems, despite their growing importance in agricultural transformation. Second, we contribute to studies examining farmer behavior around agricultural chemical use [2,39,35]. Our findings highlight how information constraints and adaptation to treatment failure can lead to unintended, environmentally damaging practices. Finally, we extend the literature on livestock health management in developing countries [9,40,17]. While previous work has focused primarily on the technical aspects of tick resistance or farmer knowledge and attitudes, we provide novel evidence linking these practices to quantifiable environmental and health risks.

In addition, our investigation also provides methodological advancements for examining the impacts of livestock systems on the environment. By adapting the environmental impact quotient (EIQ) framework – previously applied primarily to crop systems – developing a quantitative assessment of environmental and human health risks associated with livestock disease management practices in a developing country context. This approach could prove valuable for future research examining environmental and human health trade-offs in livestock intensification.

## Conclusion

This paper contributes to the growing literature on the environmental and human health implications of agricultural intensification in developing countries by examining acaricide use practices in dairy farming systems. While previous research has extensively documented the environmental impacts of pesticide use in crop production, our study provides novel evidence on the environmental and human health risks associated with chemical-based vector control in livestock systems, using Kenya’s dairy sector as an empirical setting.

Our analysis reveals several important findings. Descriptively, the study documents widespread suboptimal acaricide practices: 20% of farmers engage in improper rotation of active ingredients, 66% under-apply acaricides relative to recommended application rates, and about 29% report adverse health effects despite 65% using protective gear. More concerning is the emergence of potentially hazardous adaptation strategies, including the use of crop pesticides for tick control and the admixing of different chemical products to enhance perceived efficacy. Our 2SLS estimation provides empirical evidence that improper acaricide rotation significantly amplifies environmental and human health risks with each additional acaricide product in a farmer’s annual rotation increasing the environmental and human health risks by 26%.

### Recommendation

These findings have important policy implications for animal health management in SSA and other developing countries. First, they highlight an urgent need to reform current approaches to animal disease control, balancing the economic imperatives of livestock productivity with environmental and public health considerations. Agricultural extension services should be strengthened to improve farmers’ knowledge of proper acaricide rotation and application practices, particularly focusing on the risks of improper active ingredient rotation and chemical mixing. Second, our results suggest that policy interventions targeting agro-veterinary shops could be particularly effective, as these represent crucial last-mile information sources for farmers. However, this would require enhanced regulation of agro-veterinary services to ensure quality advice from trained professionals.

### Study limitations

It is important to note a few limitations in this study. First, the study relies on cross-sectional data and does not adequately account for time-varying factors that may influence our outcomes in the long run, and may be difficult to fully account for all possible sources of heterogeneity. Hence, our estimations remain associational. Second, the main outcome variable used is based on recall data, which is prone to measurement errors. Looking ahead, future research should explore several promising directions. Studies could evaluate the cost-effectiveness of alternative tick control strategies, including biological controls and vaccination programs, accounting for both productivity and environmental impacts. Additionally, research is needed to understand the long-term environmental accumulation of acaricide residues in different production systems and their implications for ecosystem health. Finally, given the role of information constraints in driving suboptimal practices, experimental studies could test different approaches to improving farmers’ knowledge and adoption of safer acaricide management practices.

## Supporting information

S1 File S1-S4 Tables of EIQ and full regression results. (DOCX)

## References

[1] Tran L, Skevas T, McCann L. Measuring pesticide overuse and its determinants: Evidence from Vietnamese rice and fruit farms. Aus J Agri Res Econ. 2023;67(3):417–37. doi: 10.1111/1467-8489.12521

[2] Schreinemachers P, Grovermann C, Praneetvatakul S, Heng P, Nguyen TTL, Buntong B. How much is too much? Quantifying pesticide overuse in vegetable production in Southeast Asia. J Clean Prod. 2020;244.

[3] Meunier E, Smith P, Griessinger T, Robert C. Understanding changes in reducing pesticide use by farmers: Contribution of the behavioural sciences. Agricult Syst. 2024;214.

[4] Maina KW, Parlasca MC, Rao EJO, Qaim M. Farmer‐friendly delivery of veterinary services: Experimental insights from the Kenyan dairy sector. J Agric Econ. 2024;75(3).

[5] Teufel N, Korir L, Hammond J, van Wijk M, Kiara H. Farm and Livelihood Characteristics After ITM Vaccination Against East Coast Fever in Tanzania. Front Vet Sci. 2021;8:639762. doi: 10.3389/fvets.2021.639762 34859079 PMC8632140

[6] Nagagi YP, Kimaro EG, Temba V. Practical application and the possible emergence of tick resistance to commonly used acaricides in various districts of Tanzania. Livest Res Rural Dev. 2020;32(8).

[7] Thullner F, Willadsen P, Kemp D. Acaricide rotation strategy for managing resistance in the tick Rhipicephalus (Boophilus) microplus (Acarina: Ixodidae): Laboratory experiment with a field strain from Costa Rica. J Med Entomol. 2007;44(5). 10.1603/0022-2585(2007)44[817:arsfmr]2.0.co;2 17915514

[8] Abbas RZ, Zaman MA, Colwell DD, Gilleard J, Iqbal Z. Acaricide resistance in cattle ticks and approaches to its management: the state of play. Vet Parasitol. 2014;203(1–2):6–20. 24709006 10.1016/j.vetpar.2014.03.006

[9] Vudriko P, Okwee-Acai J, Byaruhanga J, Tayebwa DS, Okech SG, Tweyongyere R, et al. Chemical tick control practices in southwestern and northwestern Uganda. Ticks Tick Borne Dis. 2018;9(4):945–55. doi: 10.1016/j.ttbdis.2018.03.009 29606621

[10] Mutavi F, Heitkönig I, Wieland B, Aarts N, Van Paassen A. Tick treatment practices in the field: Access to, knowledge about, and on-farm use of acaricides in Laikipia, Kenya. Ticks Tick Borne Dis. 2021;12(5):101757. doi: 10.1016/j.ttbdis.2021.101757 34147920

[11] Benard O O. Lessonsi Sustainable Dairy Farming to Kenyan Dairy Sector from the Dutch Dairy Sector. J Adv Dairy Res. 2016;04(04). doi: 10.4172/2329-888x.1000162

[12] Lukuyu BA, Franzel S, Ongadi P. Livestock feed resources: Current production and management practices in central and northern rift valley provinces of Kenya. Livest Res Rural Dev. 2018;23(5).

[13] Chepkwony R, Castagna C, Heitkönig I, van Bommel S, van Langevelde F. Associations between monthly rainfall and mortality in cattle due to East Coast fever, anaplasmosis and babesiosis. Parasitology. 2020;147(14):1743–51. doi: 10.1017/S0031182020001638 32907657 PMC10317715

[14] Muyobela J, Nkunika POY, Mwase ET. Resistance status of ticks (Acari; Ixodidae) to amitraz and cypermethrin acaricides in Isoka District, Zambia. Trop Anim Health Prod. 2015;47(8). 10.1007/s11250-015-0906-4 26310511

[15] Githaka NW, Kanduma EG, Wieland B, Darghouth MA, Bishop RP. Acaricide resistance in livestock ticks infesting cattle in Africa: Current status and potential mitigation strategies. Curr Res Parasitol Vect-Borne Dis. 2022;2. 10.1016/j.crpvbd.2022.100090 PMC9160480 35664895

[16] De Meneghi D, Stachurski F, Adakal H. Experiences in tick control by acaricide in the traditional cattle sector in Zambia and Burkina Faso: Possible environmental and public health implications. Front Publ Health. 2016;4. 10.3389/fpubh.2016.00239 PMC5101216 27882313

[17] Bishop RP, Githaka NW, Bazarusanga T, Bhushan C, Biguezoton A, Vudriko P, et al. Control of ticks and tick-borne diseases in Africa through improved diagnosis and utilisation of data on acaricide resistance. Parasit Vectors. 2023;16(1):224. doi: 10.1186/s13071-023-05803-3 37415211 PMC10327166

[18] Rojas-Cabeza JF, Moreno-Cordova EN, Ayala-Zavala JF, Ochoa-Teran A, Sonenshine DE, Valenzuela JG. A review of acaricides and their resistance mechanisms in hard ticks and control alternatives with synergistic agents. Acta Trop. 2025;261:107519. 39746593 10.1016/j.actatropica.2024.107519 PMC11729571

[19] Miyama T, Byaruhanga J, Okamura I, Uchida L, Muramatsu Y, Mwebembezi W, et al. Effect of chemical tick control practices on tick infestation and Theileria parva infection in an intensive dairy production region of Uganda. Ticks Tick Borne Dis. 2020;11(4):101438. doi: 10.1016/j.ttbdis.2020.101438 32299787

[20] Laing G, Aragrande M, Canali M, Savic S, De Meneghi D. Control of Cattle Ticks and Tick-Borne Diseases by Acaricide in Southern Province of Zambia: A Retrospective Evaluation of Animal Health Measures According to Current One Health Concepts. Front Public Health. 2018;6:45. doi: 10.3389/fpubh.2018.00045 29637063 PMC5881173

[21] Groot MJ, Van’t Hooft KE. The Hidden Effects of Dairy Farming on Public and Environmental Health in the Netherlands, India, Ethiopia, and Uganda, Considering the Use of Antibiotics and Other Agro-chemicals. Front Public Health. 2016;4:12. doi: 10.3389/fpubh.2016.00012 26942171 PMC4764701

[22] Rani L, Thapa K, Kanojia N, Sharma N, Singh S, Grewal AS, et al. An extensive review on the consequences of chemical pesticides on human health and environment. J Clean Product. 2021;283.

[23] Njuki J, Poole EJ, Johnson NL, Baltenweck I, Pali PN, Lokman Z, et al. Gender, livestock and livelihood indicators [Internet]. Nairobi: International Livestock Research Institute; 2011 Aug [cited 2023 Aug 24]. Report No.: Version 2. Available from: https://hdl.handle.net/10568/33974

[24] Kouser S, Qaim M. Valuing financial, health, and environmental benefits of Bt cotton in Pakistan. Agricult Econ. 2013;44(3):323–35. doi: 10.1111/agec.12014

[25] Kovach J, Petzoldt C, Degni J, Tette J. A method to measure the environmental impact of pesticides. New York’s Food Life Sci Bullet. 1992;139(139).

[26] Eshenaur B, Grant J, Kovach J, Petzoldt C, Degni J, Tette J. Vol. 2015, New York state integrated pest management program, cornell cooperative extension, Cornell University. 1992-2015. Environmental Impact Quotient: A method to measure the environmental impact of pesticides. 2015 [cited 2024 Mar 18]. Available from: https://nysipm.cornell.edu/eiq/calculator-field-use-eiq

[27] Midingoyi S, Kifouly G, Kassie M, Muriithi B, Diiro G, Ekesi S. Do farmers and the environment benefit from adopting integrated pest management practices? Evidence from Kenya. J Agric Econ. 2019;70(2):452–70.

[28] Schreinemachers P, Sringarm S, Sirijinda A. The role of synthetic pesticides in the intensification of highland agriculture in Thailand. Crop Protect. 2011;30(11):1430–7. doi: 10.1016/j.cropro.2011.07.011

[29] Oliver DP, Kookana RS, Miller RB, Correll RL. Comparative environmental impact assessment of herbicides used on genetically modified and non-genetically modified herbicide-tolerant canola crops using two risk indicators. Sci Total Environ. 2016;557–558:754–63. doi: 10.1016/j.scitotenv.2016.03.106 27039064

[30] Stock JH, Yogo M. Testing for weak instruments in linear IV regression. Cambridge (MA): National Bureau of Economic Research; 2002.

[31] Angrist JD, Pischke JS. Mostly harmless econometrics: An empiricist’s companion. Princeton University Press; 2009.

[32] Kripfganz S, Kiviet JF. Kinkyreg: Instrument-free inference for linear regression models with endogenous regressors. Stata J. 2021;21(3).

[33] Tabe-Ojong MP. Context matters: Oil palm production and women’s dietary diversity in the tropical forest of Cameroon. J Agric Econ. 2024;75(1).

[34] World Health Organization WHO. The WHO recommended classification of pesticides by hazard and guidelines to classification 2019. World Health Organization; 2020.

[35] Zhang C, Hu R, Shi G, Jin Y, Robson MG, Huang X. Overuse or underuse? An observation of pesticide use in China. Sci Total Environ. 2015;538:1–6. doi: 10.1016/j.scitotenv.2015.08.031 26296070

[36] Van Hoi P, Mol A, Oosterveer P. State governance of pesticide use and trade in Vietnam. NJAS. 2013;67(1):19–26. doi: 10.1016/j.njas.2013.09.001

[37] Sheahan M, Barrett CB, Goldvale C. Human health and pesticide use in Sub‐Saharan Africa. Agricultural Economics. 2017;48(S1):27–41. doi: 10.1111/agec.12384

[38] Kim K-H, Kabir E, Jahan SA. Exposure to pesticides and the associated human health effects. Sci Total Environ. 2017;575:525–35. doi: 10.1016/j.scitotenv.2016.09.009 27614863

[39] Ghimire N, Woodward RT. Under- and over-use of pesticides: An international analysis. Ecol Econ. 2013;89:73–81. doi: 10.1016/j.ecolecon.2013.02.003

[40] Marsh TL, Yoder J, Deboch T, McElwain TF, Palmer GH. Livestock vaccinations translate into increased human capital and school attendance by girls. Sci Adv. 2016;2(12):e1601410. doi: 10.1126/sciadv.1601410 27990491 PMC5156515

