# Supplementary material for: From protection to pollution: Evaluating environmental and human health risks of acaricide use in dairy farming in Kenya
Source: PLoS One. 2025 Oct 17;20(10):e0333694. doi: 10.1371/journal.pone.0333694 (PMC12533840; doi:10.1371/journal.pone.0333694)
Supplement: S1 File — (DOCX) [file pone.0333694.s001.docx]

**Online supplementary material**

| **S1 Table. EIQ values for different acaricides by active ingredients.** | | | | |
| --- | --- | --- | --- | --- |
| **Active ingredient** | **EIQ farm worker** | **EIQ consumer** | **EIQ ecology** | **EIQ value** |
| Amitraz 12.5% | 27 | 2.5 | 46 | 25.17 |
| Alpha-cypermethrin 10% | 6 | 3 | 71 | 26.67 |
| Lambdacyhalothrin 1.75% | 13.11 | 4.99 | 114.04 | 44.05 |
| Cyhalothrin 5% | 20.7 | 3.45 | 108.35 | 44.17 |
| Cypermethrin 10% | 13.8 | 5.9 | 89.35 | 36.35 |
| Deltamethrin 2.5% | 18 | 2 | 65.15 | 28.38 |
| Chlorfenvinphos 30% | 65.55 | 7.66 | 93.53 | 55.58 |
| Chlorpyrifos 50% + Cypermethrin 5% | - | - | - | - |
| Chlorpyrifos 50% | 6 | 2 | 72.55 | 26.85 |
| Cypermethrin 1.5% + Chlorpyrifos 2.5% + Piperonyl butoxide 1.5%+ Citronell 0.1% | - | - | - | - |
| Piperonyl butoxide 1.5% | 10.35 | 4.15 | 62.82 | 25.77 |
| Citronell 0.1% | NA | NA | NA | NA |

| **S2 Table. Mean EIQ field use by acaricide group.** | |
| --- | --- |
| **Acaricide group** | **Mean EIQ field use** |
| Formamidines | 36.09 |
| Pyrethroids | 23.23 |
| Organophosphate | 216.73 |
| Combinations | 178.04 |

| **S3 Table. First-stage coefficient estimates for the determinants of the number of acaricides used annually.** | |
| --- | --- |
| **Controls** | **Improper acaricide rotation** |
| Male household head (male = 1) | 0.02 |
|  | (0.11) |
| Dairy farming experience (years) | -0.01** |
|  | (0.00) |
| Household head main occupation (farming = 1) | 0.06 |
|  | (0.10) |
| Grazing system (yes = 1) | 0.08 |
|  | (0.09) |
| Livestock ownership (TLU) | 0.04*** |
|  | (0.03) |
| Log annual household expenditure (KES) | 0.01 |
|  | (0.03) |
| Number of extension visits (annually) | 0.00 |
|  | (0.00) |
| Use protective gear (yes = 1) | 0.02 |
|  | (0.10) |
| Distance to nearest vet shop (kilometers) | 0.00 |
|  | (0.02) |
| Constant | 1.74*** |
|  | (0.42) |
| ***Instrument*** |  |
| Incidence of improper acaricide rotation (yes = 1) | 1.34*** |
|  | (0.12) |
| R-squared | 0.32 |
| *F* statistic | 19.09 |
| Observations | 412 |

Notes: Regression coefficients are shown with robust standard errors in parenthesis. Statistical significance at *p < 0.1, **p < 0.05, ***p < 0.01.

| **S4 Table. Second-stage coefficient estimates for the association between improper acaricide rotation and EIQ field use.** | |
| --- | --- |
| **Controls** | **Log EIQ field use** |
| Number of acaricides used annually | 0.26*** |
|  | (0.08) |
| Male household head (male = 1) | 0.01 |
|  | (0.11) |
| Dairy farming experience (years) | -0.00 |
|  | (0.00) |
| Household head main occupation (farming = 1) | -0.15 |
|  | (0.10) |
| Grazing system (yes = 1) | -0.07 |
|  | (0.09) |
| Livestock ownership (TLU) | -0.06** |
|  | (0.02) |
| Log annual household expenditure (KES) | -0.11** |
|  | (0.05) |
| Number of extension visits (annually) | -0.01** |
|  | (0.00) |
| Use protective gear (yes =1) | -0.15 |
|  | (0.10) |
| Distance to nearest vet shop (kilometers) | 0.00 |
|  | (0.02) |
| Constant | 8.95*** |
|  | (0.67) |
| Observations | 412 |

Notes: Regression coefficients are shown with robust standard errors in parenthesis. Statistical significance at *p < 0.1, **p < 0.05, ***p < 0.01.
